# Hyperperfusion of bilateral amygdala in patients with chronic migraine: an arterial spin-labeled magnetic resonance imaging study

**DOI:** 10.1186/s10194-023-01668-0

**Published:** 2023-10-18

**Authors:** Xiaoyan Bai, Wei Wang, Xueyan Zhang, Zhangxuan Hu, Xue Zhang, Yingkui Zhang, Hefei Tang, Yaqing Zhang, Xueying Yu, Ziyu Yuan, Peng Zhang, Zhiye Li, Xun Pei, Yonggang Wang, Binbin Sui

**Affiliations:** 1 Tiantan Neuroimaging Center for Excellence, China National Clinical Research Center for Neurological Diseases, Beijing Tiantan Hospital and Beijing Neurosurgical Institute, Capital Medical University, No.119 South Fourth Ring West Road, Fengtai District, Beijing, 100070 China; 2 Department of Radiology, Beijing Tiantan Hospital, Capital Medical University, Beijing, China; 3 Headache Center, Department of Neurology, Beijing Tiantan Hospital, Capital Medical University, No.119 South Fourth Ring West Road, Fengtai District, Beijing, 100070 China; 4 Department of Neurology, The First Affiliated Hospital of Zhengzhou University, Zhengzhou, China; 5 GE Healthcare, Beijing, China

**Keywords:** Chronic migraine, Episodic migraine, Amygdala, Perfusion, Arterial spin labeling

## Abstract

**Background:**

Amygdala, an essential element of the limbic system, has served as an important structure in pain modulation. There is still a lack of clarity about altered cerebral perfusion of amygdala in migraine. This study aimed to investigate the perfusion variances of bilateral amygdala in episodic migraine (EM) and chronic migraine (CM) using multi-delay pseudo-continuous arterial spin-labeled magnetic resonance imaging (pCASL-MRI).

**Methods:**

Twenty-six patients with EM, 55 patients with CM (33 CM with medication overuse headache (MOH)), and 26 age- and sex-matched healthy controls (HCs) were included. All participants underwent 3D multi-delay pCASL MR imaging to obtain cerebral perfusion data, including arrival-time-corrected cerebral blood flow (CBF) and arterial cerebral blood volume (aCBV). The CBF and aCBV values in the bilateral amygdala were compared among the three groups. Correlation analyses between cerebral perfusion parameters and clinical variables were performed.

**Results:**

Compared with HC participants, patients with CM were found to have increased CBF and aCBV values in the left amygdala, as well as increased CBF values in the right amygdala (all *P* < 0.05). There were no significant differences of CBF and aCBV values in the bilateral amygdala between the HC and EM groups, the EM and CM groups, as well as the CM without and with MOH groups (all *P* > 0.05). In patients with CM, the increased perfusion parameters of bilateral amygdala were positively correlated with MIDAS score after adjustments for age, sex, and body mass index (BMI).

**Conclusion:**

Hyperperfusion of bilateral amygdala might provide potential hemodynamics evidence in the neurolimbic pain network of CM.

**Supplementary Information:**

The online version contains supplementary material available at 10.1186/s10194-023-01668-0.

## Introduction

Migraine is the second leading cause of disability worldwide, with a 1-year prevalence of 15% in the general population; it has significant impacts on socioeconomic function and quality of life in affected patients [1, 2]. Most people with migraine have episodic migraine (EM), which is defined as < 15 days of headache per month. Some people with migraine have chronic migraine (CM), which is defined ≥ 15 days of headache per month for > 3 months; at least eight days of headache per month must meet the criteria for migraine headaches [3]. Each year, approximately 2.5% of patients with EM progress to CM [4]. Moreover, about 30%–50% of patients with CM display medication overuse upon presentation to a headache specialist center [5]. Understanding the pathophysiological mechanism of EM and CM may provide some help for early diagnosis and treatment of migraine. A previous study favored a “neurolimbic” model of migraine, whereby brainstem pain-modulating circuits centered on periaqueductal gray matter have bidirectional connections with limbic components such as the amygdala [6]. An animal experiment showed that the basolateral amygdala–prefrontal cortex–periaqueductal gray–spinal cord pathway is the neuronal circuit that activates descending modulation of neuropathic pain [7]. As mentioned above, the amygdala, an essential part of the limbic system, has emerged as a key brain structure involved in pain modulation [7–9]. However, the rigorous neuromechanism of amygdala in episodic migraine and migraine chronicization remains unknown.

Brain structural and resting-state functional magnetic resonance imaging (rs-fMRI) of the amygdala has been increasingly used to investigate the neuromechanisms underlying CM. Using a voxel-based morphometry approach, Neeb et al. [10] detected increased gray matter volume in the amygdala and putamen in patients with CM, compared with healthy controls (HCs). Another resting-state functional MRI study explored changes in amygdala functional connectivity in patients with migraine, which revealed that the neurolimbic pain network contributes to EM pathogenesis and migraine chronicization [11]. However, previous studies regarding cerebral perfusion in the amygdala have focused on individuals with psychiatric disorders [12–14]. The presence of increased CBF values in the right amygdala indicated that healthy young first-degree relatives of patients with major depression were more likely to develop depression [12]. The hemodynamic aspects of the amygdala were not explored in patients with migraine.

Arterial spin labeling (ASL) is a non-invasive cerebral perfusion MRI technique that enables quantitative assessment of brain perfusion without the use of a gadolinium-based contrast agent [15]. The reliability and reproducibility of ASL in cerebral perfusion measurement have been validated in previous studies [16–18]. Three-dimensional pseudo-continuous ASL (3D pCASL) imaging can be used for whole-brain scanning; its increasing acceptance in clinical practice is related to its ease of implementation and high signal-to-noise ratio [19]. Multi-delay pCASL, in which images with various post-label delay (PLD) times are acquired to improve the accuracy of cerebral blood flow (CBF) quantification, has been used for the assessment of ischemic stroke, moyamoya disease, and status epilepticus [20–22]. Multiple studies have revealed altered cerebral perfusion in patients with EM, along with abnormal regional hyperperfusion in gray matter [23–25]. However, to our knowledge, no study has investigated cerebral perfusion of the amygdala in patients with EM or CM using multi-delay 3D pCASL MRI. We hypothesized that the cerebral perfusion for the bilateral amygdala may change in patients with EM and CM, compared with HCs; moreover, medication overuse in patients with CM may alter cerebral perfusion levels in bilateral amygdala, compared with patients without MOH.

In this study, we investigated cerebral perfusion levels in the bilateral amygdala among patients with EM and patients with CM using multi-delay pCASL MRI. We also evaluated whether medication overuse altered cerebral perfusion of the bilateral amygdala in patients with CM.

## Materials and methods

### Participants

The present study was a substudy of the ongoing China HeadAche DIsorders RegiStry Study (CHAIRS, trial registration number: NCT05334927, https://www.clinicaltrials.gov). From October 2020 to November 2022, 28 healthy controls (HCs) and 94 patients diagnosed with EM (*n* = 30) or CM (*n* = 64) were consecutively enrolled in the Headache Center, Department of Neurology, Beijing Tiantan Hospital, Capital Medical University. This work was approved by the Institutional Review Board (KY2022-044) of Beijing Tiantan Hospital, Capital Medical University. All participants provided written informed consent prior to study enrollment. The inclusion criteria for EM and CM groups were (1) fulfillment of the criteria for EM or CM, according to the 3rd edition of the International Classification of Headache Disorders (ICHD-3) [3], (2) age 20 to 65 years, (3) all patients with migraine without aura. The exclusion criteria for EM and CM groups were (1) presence of another type of primary headache, (2) MRI claustrophobia or contraindications, (3) poor image quality, and (4) brain damage or other neurological diseases (e.g., epilepsy, stroke, and/or physical disease) that could affect the results of the study. The inclusion criteria for HCs were (1) ability to undergo MRI scanning (i.e., no claustrophobia and no metal in the body), (2) no neurological or other major systemic diseases, and (3) age and sex matched to patients in the EM and CM groups. The exclusion criteria for HCs were (1) pregnancy or breastfeeding, (2) MRI contraindications, and (3) poor MRI data quality.

Demographic data were recorded for all participants. Clinical assessment scales including the Headache Impact Test-6 (HIT-6), Patient Health Questionnaire-9 (PHQ-9), Generalized Anxiety Disorder-7 (GAD-7), and Pittsburgh Sleep Quality Index (PSQI) were administered by an experienced neurologist and recorded as components of our headache questionnaire, prior to magnetic resonance data acquisition [26]. All participants had not taken medication before the end of MR scan and didn’t suffer discomfort during the MRI scans.

### MRI data acquisition

MRI was performed on a 3T MR scanner (Signa Premier, GE Healthcare, Waukesha, WI, USA) using a 48-channel head coil. All participants were instructed to lie in a supine position, and foam padding was used to limit head movement. Three-dimensional T1-weighted anatomical images were acquired using the magnetization- prepared rapid gradient- echo (MP-RAGE) sequence with 1.0-mm isotropic resolution. The following parameters were used: repetition time = 400 ms, echo time = 3 ms, slice thickness = 1 mm, slice number = 192, flip angle = 8°, field of view = 256 × 256 mm^2^, reconstruction matrix = 256 × 256, acceleration factor = 2, and acquisition time = 4 min. Volumetric perfusion imaging was conducted using a multi-delay pCASL sequence with spiral readout. The following parameters were used: repetition time = 7138 ms, echo time = 11 ms, in-plane spatial resolution = 4.1 mm × 4.1 mm, slice thickness = 4.5 mm, NEX = 1, readout = 5 arms × 640 samples, field of view = 208 mm × 208 mm, and reconstruction matrix = 128 × 128. The total examination time for the ASL protocol was 4 min 44 s. This protocol encodes seven different post-labeling delay (PLD) times into a single acquisition. Images were acquired with PLD times of 1.00, 1.36, 1.74, 2.14, 2.57, 3.07, and 3.66 s, as well as effective label durations (LD) of 0.36, 0.38, 0.40, 0.44, 0.49, 0.59, and 0.84 s.

### Imaging analysis

Arterial transit time (ATT) maps were estimated with signal weighted delay, as described by Dai et al. [27]. For each pair of PLD and LD, the arrival-time-corrected CBF maps were quantified as follows:

$$CBF= \frac{6000{e}^{\delta /{T}_{1a}}}{2\epsilon {T}_{1a}({e}^{-\frac{\mathrm{max}\left(\omega -\delta ,0\right)}{{T}_{1a}}}-{e}^{-\frac{\mathrm{max}\left(\tau +\omega -\delta ,0\right)}{{T}_{1a}}})}\frac{M}{M0},$$

where $\delta$ is the ATT, $\tau$ is the LD, $\omega$ is the PLD, ${T}_{1a}$ is the longitudinal relaxation time of arterial blood (1.6 s), $\epsilon$ is the combined efficiency of labeling and background suppression (0.63), $M$ is the signal intensity of the perfusion weighted image, and M0 is the signal intensity of the reference image. The final CBF was the mean of the estimated CBF at each pair of PLD and LD. Arterial cerebral blood volume (aCBV) maps were generated by the product of ATT and CBF, which represents arterial blood volume from the labeling plane to the imaging voxel [28]:

$$aCBV=CBF\cdot ATT$$

**Fig. 1:**
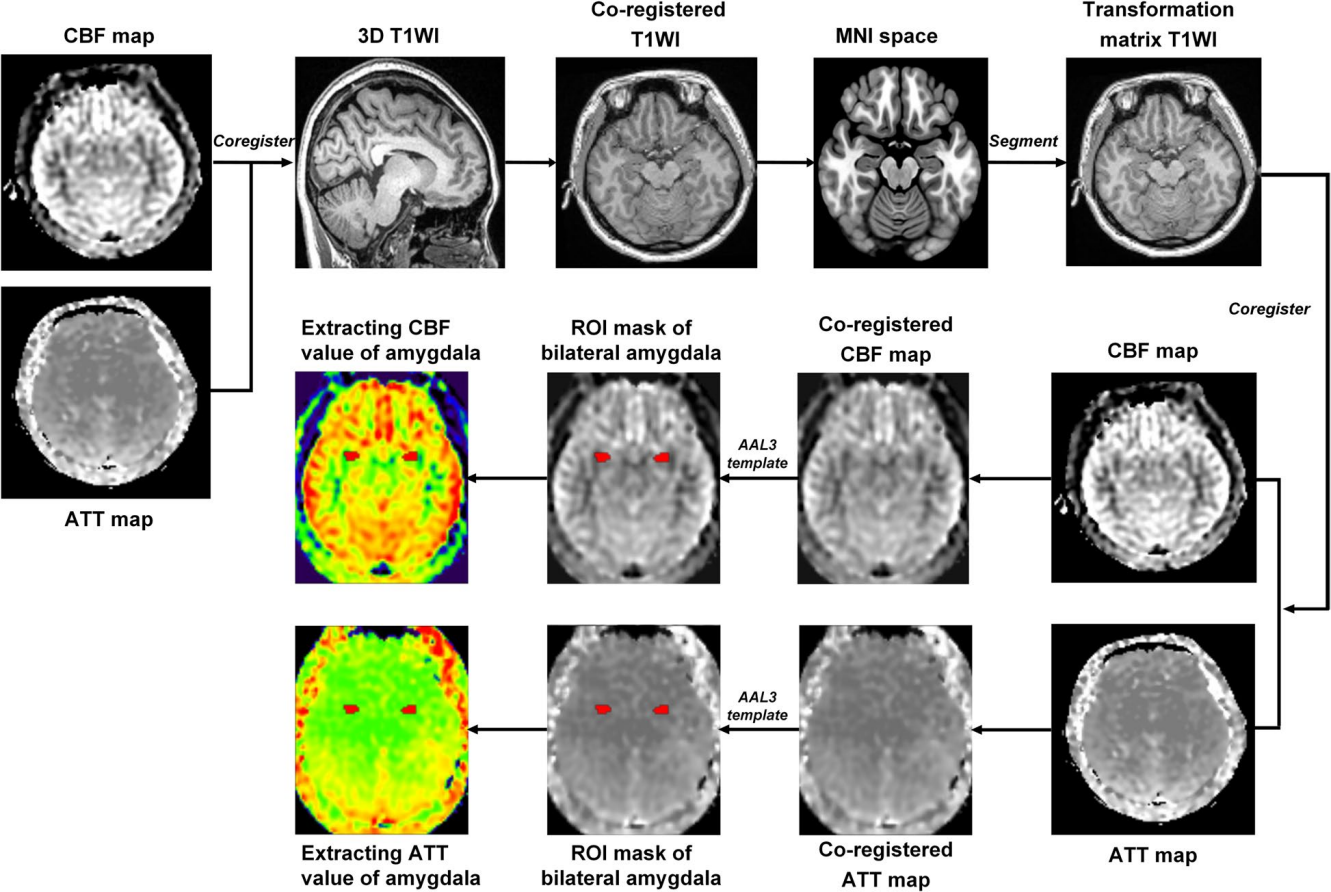
Post-processing diagram of cerebral perfusion by multi-delay ASL MR imaging in bilateral amygdala. Firstly, the 3D T1 image was respectively co-registered to the arrival-time-corrected cerebral blood flow (CBF) and arterial transit time (ATT) maps. Then the CBF and ATT maps were linearly co-registered in the native space to their corresponding 3D T1WI images, which were non-linearly registered to the standard Montreal Neurologic Institute (MNI) stereotaxic space. Finally, the co-registered CBF and ATT images were generated. The ROI mask of the amygdala was generated by the automatic anatomical labeling atlas 3 (AAL3) template. The CBF and ATT values of amygdala were extracted from the registered CBF and ATT images based on the amygdala mask

ASL data were preprocessed with Statistical Parametric Mapping 12 (SPM 12) software (http://www.fil.ion.ucl.ac.uk/spm/) using Matlab (Mathworks Inc., Natick, MA). Arrival-time-corrected CBF and ATT maps were linearly co-registered in the native space to their corresponding 3D T1-weighted images, which were non-linearly registered to the standard Montreal Neurologic Institute (MNI) stereotaxic space. The transformation matrix converting T1-weighted structural images to MNI space were further used to transform the corresponding arrival-time corrected CBF and ATT maps to MNI space. The automated anatomical labelling atlas 3 (AAL3) [29] was used to parcellate and generate bilateral amygdala masks. All bilateral amygdala masks were overlaid on the co-registered CBF and ATT maps. Subsequently, the CBF and ATT values of bilateral amygdala were extracted from the registered CBF and ATT images based on the amygdala masks. Finally, the mean CBF, ATT, and aCBV values of bilateral amygdala were calculated (Fig. 1).

### Statistical analysis

The sample size was based on the previous research and available data in this study. A minimum sample size of 32 (16 HCs and 16 patients with EM) was expected to provide 80% power to reject the null hypothesis of equal means, assuming a mean difference of 7.8 (40.9–33.1) with standard deviations of 5.9 for the HC group and 9.4 for the EM group (two-sided alpha = 0.05) [25]. Considering an anticipated dropout rate of 20%, the total sample size required was 38 participants (19 HCs and 19 patients with EM). Furthermore, a minimum sample size of 30 participants (15 HCs and 15 patients with CM) was expected provide 80% power to reject the null hypothesis of equal means, assuming a mean difference of 5.49 (55.83–49.34) with standard deviations of 6.55 for the HC group and 6.09 for the CM group (two-sided alpha = 0.05) [30]. Considering an anticipated dropout rate of 20%, the total sample size required was 36 participants (18 HCs and 18 patients with CM). Accordingly, 26 HCs, 26 patients with EM, and 55 patients with CM were included in this study. All statistical analyses were performed using SPSS 26.0 software (SPSS Inc., Chicago, IL, USA). The Kolmogorov–Smirnov test was used to assess the normality of clinical data and cerebral perfusion parameters. All quantitative data were expressed as means ± standard deviations for normally distributed data or medians with ranges for non-normally distributed data. Categorical variables were analyzed using the chi-squared test or Fisher’s exact test. Differences in clinical characteristics between the EM and CM groups, as well as the CM with and without medication overuse headache (MOH) groups, were analyzed using independent samples t-tests for normally distributed data and the Mann–Whitney U test for non-normally distributed data. For normally distributed data, comparisons of CBF and aCBV values in the bilateral amygdala among the three groups (HC, EM, and CM) were performed by one-way analysis of variance; post hoc analysis with Bonferroni correction was used for multiple comparisons. For non-normally distributed data, comparisons of CBF and aCBV values in the bilateral amygdala among the three groups were performed by the Kruskal–Wallis H test; all pairwise comparisons were performed using Kruskal–Wallis one-way analysis of variance (k samples). Comparisons of CBF and aCBV values in the amygdala between the CM with and without MOH groups were performed using independent samples t-tests or the Mann–Whitney U test, according to the normality of the data distribution; comparisons of CBF and aCBV values between the left and right sides of the amygdala in the HC, EM and CM groups were conducted using the same tests. Correlations between clinical characteristics and the perfusion parameters of the bilateral amygdala were determined using partial correlation analysis with age, sex, and BMI as covariates. Positive values of the correlation coefficient *r* represented positive correlations. Two-sided *P*-values < 0.05 were considered statistically significant.

## Results

### Demographic and clinical characteristics

**Fig. 2:**
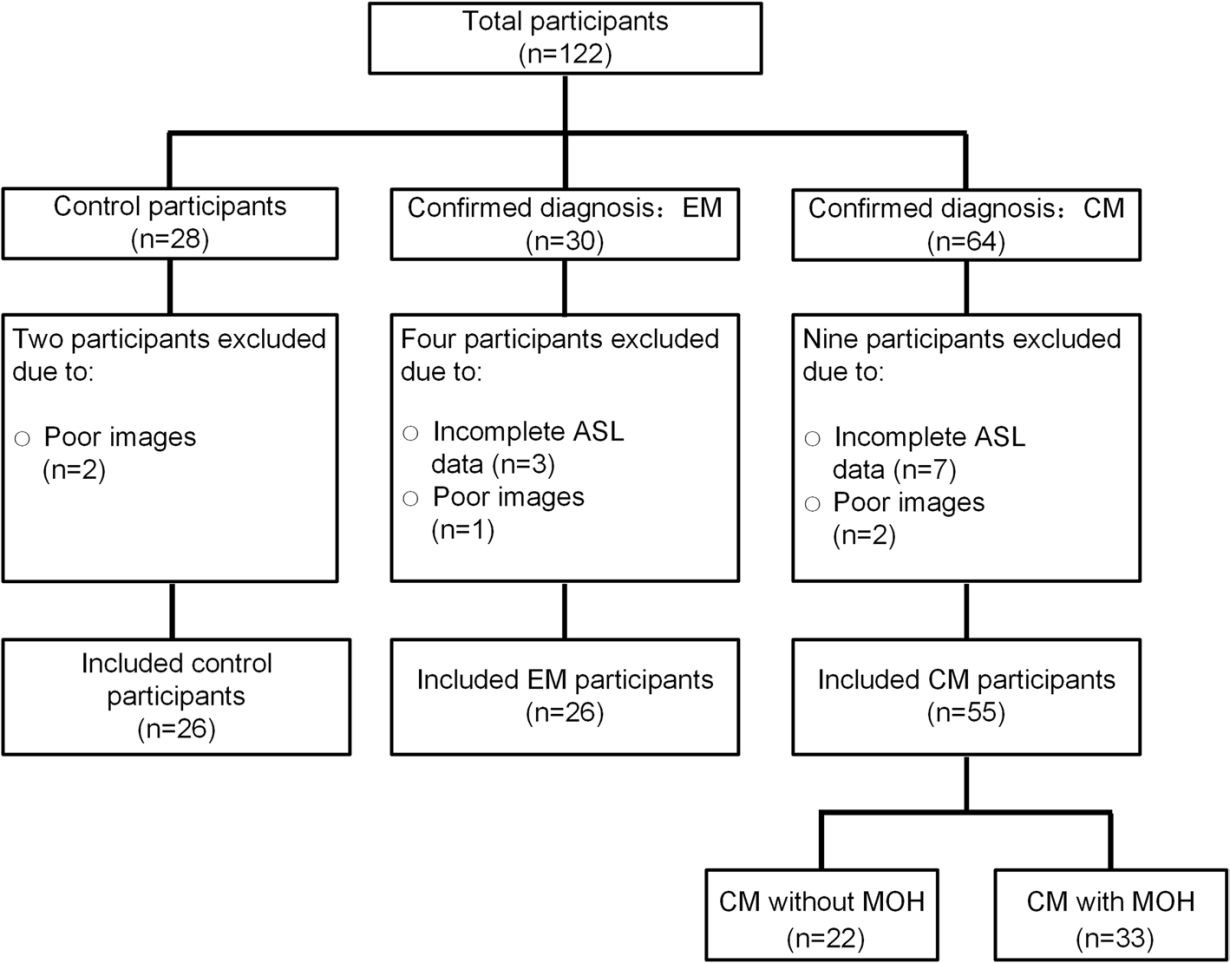
The patient enrollment flowchart

One hundred twenty-two participants (28 age- and sex-matched HCs, 30 patients with EM, and 64 patients with CM) were consecutively recruited in this study. The patient enrollment flowchart is shown in Fig. 2. Four patients with EM were excluded because of incomplete ASL data (*n* = 3) and poor imaging quality (*n* = 1). Nine patients with CM were excluded because of incomplete ASL data (*n* = 7) and poor imaging quality (*n* = 2). Two HCs were excluded because of poor imaging quality (*n* = 2). In total, 107 participants, including 26 HCs, 26 patients with EM, and 55 patients with CM, were included in this study. The demographic and clinical characteristics of the three groups are summarized in Table 1. All participants were right-handed. The three groups did not differ in terms of age, sex, or body mass index (BMI). Compared with patients who had EM, patients with CM displayed longer disease duration (*P* = 0.025), and greater headache frequency (*P* < 0.001). Additionally, patients with CM had higher scores on the Migraine Disability Assessment (MIDAS) (*P* < 0.001), PHQ-9 (*P* = 0.001), and GAD-7 (*P* = 0.005), compared with patients who had EM.

**Table 1:** Demographic and clinical characteristics among HC, EM, and CM

	**HC (*n* = 26)**	**EM (*n* = 26)**	**CM (*n* = 55)**	***P* value**
Age (years)	41.50 (31.75–46.25)	39.50 (32.00–46.50)	46.00 (33.75–52.00)	0.201
Female, n (%)	19 (73.1)	19 (73.1)	43 (78.2)	0.828
BMI (kg/m^2^)	22.72 (20.05–25.00)	22.82 (21.10–26.15)	22.87 (20.94–25.41)	0.723
Right-handers, n (%)	26 (100.0)	26 (100.0)	55 (100.0)	1.000
Unilateral headache, n (%)	NA	10 (38.5)	25 (45.5)	0.553
Disease duration (years)	NA	15.48 ± 10.00	21.00 ± 10.67	0.025^*^
Headache frequency, days/month	NA	8.00 (4.00–12.75)	25.00 (15.00–30.00)	< 0.001^***^
Headache intensity^a^	NA	7.00 (6.25–8.00)	7.00 (6.00–9.00)	0.221
MIDAs score (0–270)	NA	50.00 (18.00–70.00)	100.00 (59.00–179.00)	< 0.001^***^
HIT-6 score (36–78)	NA	64.00 (59.25–68.75)	66.00 (64.00–71.75)	0.090
PHQ-9 score (0–27)	NA	4.00 (1.00–7.00)	8.50 (3.75–16.00)	0.001^**^
GAD-7 score (0–21)	NA	2.00 (1.00–4.50)	6.00 (2.00–13.25)	0.005^**^
PSQI score (0–21)	NA	8.30 ± 4.54	10.15 ± 4.67	0.122

*HC* healthy control, *EM* episodic migraine, *CM* chronic migraine, *BMI* body mass index, *MIDAs* Migraine Disability Assessment Scale, *HIT-6* Headache Impact Test-6, *PHQ-9* Patient Health Questionnaire-9, *GAD-7* Generalized Anxiety Disorder-7, *PSQI* Pittsburgh Sleep Quality Index

^*^P < 0.05

^**^P < 0.01

^***^P < 0.001

^a^Headache intensity on a 0–10 numerical rating scale

In CM group, 33 patients with MOH and 22 without MOH were included. The demographic and clinical characteristics between the CM without and with MOH are summarized in the Supplementary Table S1. Patients who had CM with MOH displayed a higher headache frequency (*P* = 0.009) and more severe headache intensity (*P* = 0.018), compared with patients who had CM without MOH. There were no significant differences in other clinical characteristics between the two groups (all *P* > 0.05).

### Comparisons of amygdala perfusion among the HC, EM, and CM groups

As shown in Table 2, CBF and aCBV values in the left amygdala significantly differed among the HC, EM, and CM groups (*P* = 0.015 and *P* = 0.022, respectively). Moreover, CBF values in the right amygdala significantly differed among the three groups (*P* = 0.019). aCBV values in the right amygdala tended to differ among the three groups, but the extent of variation was not statistically significant (*P* = 0.053).

**Table 2:** The cerebral perfusion parameters of bilateral amygdala among different groups

	**Sides**	**HC (*n* = 26)**	**EM (*n* = 26)**	**CM (*n* = 55)**	**Effect size (η**^**2**^_*partial*_**)**	***P* value**
CBF (ml/100 g/min)	Left	40.26 ± 7.56	43.69 ± 9.00	46.50 ± 9.56	0.077	0.015^*^
	Right	38.31 (32.33–43.34)	43.35 (31.97–49.12)	42.92 (38.32–49.09)	0.083	0.019^*^
	*P* value	0.383	0.429	0.328	-	-
CBV (ml/100 g)	Left	0.79 ± 0.15	0.86 ± 0.19	0.92 ± 0.21	0.071	0.022^*^
	Right	0.79 (0.69–0.86)	0.85 (0.70–1.02)	0.86 (0.75–0.98)	0.067	0.053
	*P* value	0.692	0.846	0.384	-	-

*HC* healthy control, *EM* episodic migraine, *CM* chronic migraine, *CBF* cerebral blood flow, *aCBV* arterial cerebral blood volume

^*^P < 0.05

**Fig. 3:**
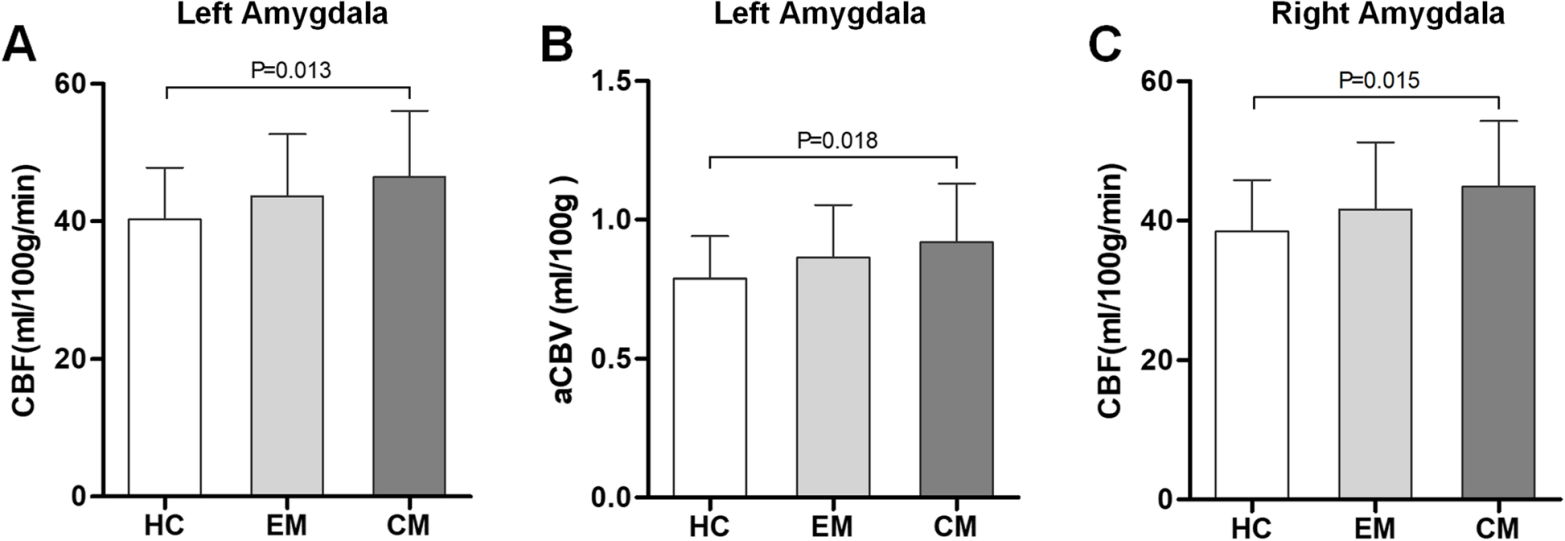
The CBF and aCBV values of the bilateral amygdala between the HC and EM groups, EM and CM groups, as well as HC and CM groups. **A**, **B** Compared to HC group, the left amygdala presented significantly increased CBF and aCBV values in CM group (*P* = 0.013 and 0.018, respectively); **C** Compared to HC group, the right amygdala showed significantly increased CBF values in CM group (*P* = 0.015). *CBF* cerebral blood flow, *aCBV* Arterial cerebral blood volume, *HC* healthy control, *EM* episodic migraine, *CM* chronic migraine

Compared with the HC group, CBF and aCBV values in the left amygdala were significantly greater (*P* = 0.013 and *P* = 0.018, respectively) in the CM group; CBF values in the right amygdala were significantly greater (*P* = 0.015) in the CM group. CBF and aCBV values in the bilateral amygdala did not significantly differ between the HC and EM groups, or between the EM and CM groups (all *P* > 0.05) (Fig. 3).

There was no significant difference in CBF and aCBV values between the left and right sides of the amygdala in the three groups (all *P* > 0.05) (Table 2).

### Comparisons of amygdala perfusion between CM without and with MOH

CBF and aCBV values in the bilateral amygdala did not significantly differ between the CM without and with MOH groups (all *P* > 0.05), as shown in Table 3. But compared to CM without MOH group, CM with MOH group exhibited relatively high CBF and CBV values in the bilateral amygdala.

**Table 3:** The cerebral perfusion parameters of bilateral amygdala between CM without and with MOH

	**Sides**	**CM (*n* = 55)**		***P* value**
		**CM without MOH (*n* = 22)**	**CM with MOH (*n* = 33)**	
CBF (ml/100 g/min)	Left	42.71 (40.63–50.08)	47.05 (38.49–52.88)	0.618
	Right	42.05 (38.04–47.20)	44.95 (39.10–52.91)	0.264
	*P* value	0.302	0.700	-
CBV (ml/100 g)	Left	0.92 ± 0.24	0.92 ± 0.20	0.975
	Right	0.81 (0.71–0.91)	0.89 (0.80–1.03)	0.164
	*P* value	0.270	0.751	-

*HC* healthy control, *CM* chronic migraine, *MOH* medication overuse headache, *CBF* cerebral blood flow, *aCBV* arterial cerebral blood volume

There was no significant difference in CBF and aCBV values between the left and right sides of the amygdala in the two groups (all *P* > 0.05) (Table 2).

### Correlation analysis between amygdala perfusion parameters and clinical characteristics

**Fig. 4:**
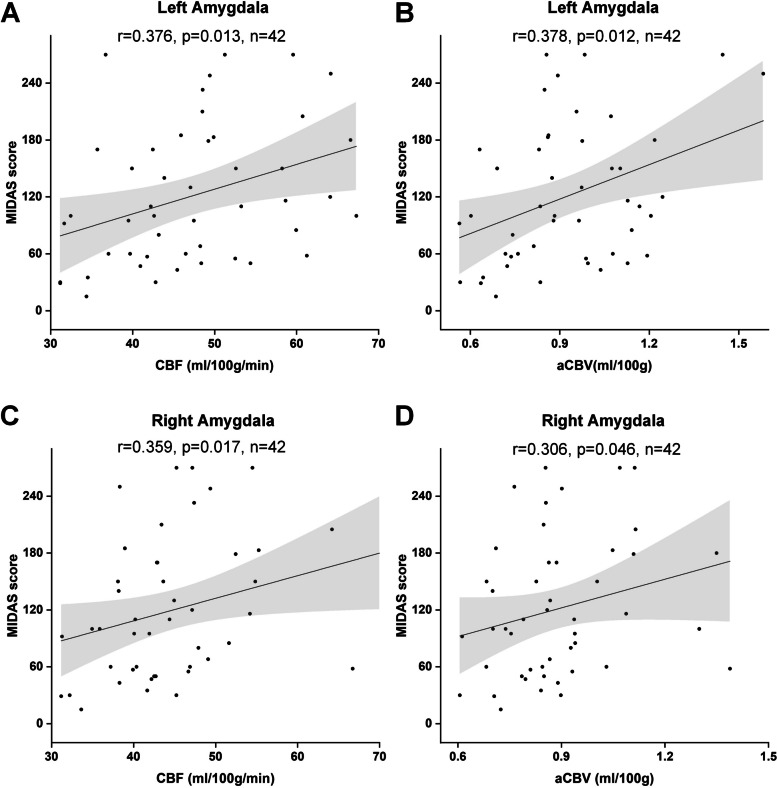
Correlation analysis between cerebral perfusion parameters and clinical variables in bilateral amygdala of CM. The CBF and aCBV values of left amygdala (**A**, **B**) and right amygdala (**C**, **D**) were positively correlated with MIDAS score. *CM* chronic migraine, *CBF* cerebral blood flow, *aCBV* Arterial cerebral blood volume, *MIDAs* Migraine Disability Assessment Scale

Among patients with CM, CBF (*r* = 0.376, *P* = 0.013, *n* = 42) and aCBV (*r* = 0.378, *P* = 0.012, *n* = 42) values in the left amygdala were positively correlated with MIDAS score; CBF (*r* = 0.359, *P* = 0.017, *n* = 42) and aCBV (*r* = 0.306, *P* = 0.046, *n* = 42) values in the right amygdala were positively correlated with MIDAS score after adjustments for age, sex and BMI (Fig. 4). Among patients with EM, there were no significant correlations between bilateral amygdala perfusion parameters and clinical characteristics (all *P* > 0.05).

## Discussion

In the present study, we found that patients with CM displayed greater cerebral perfusion in the bilateral amygdala, compared with HCs; in contrast, we found no differences in bilateral amygdala perfusion parameters between the HC and EM groups, or between the EM and CM groups. To our knowledge, this was the first study to assess cerebral perfusion levels in the bilateral amygdala among patients with EM and patients with CM based on multi-delay pCASL MR imaging. These findings were partially consistent with the results in a previous study of postherpetic neuralgia pain by Lui et al., [31] who reported a significant increase in CBF in the left amygdala among their patients. Additionally, some studies utilized cerebral perfusion imaging to explore relationships between the amygdala and psychiatric disorders. In a study of 200 healthy never-depressed first-degree relatives of patients with major depressive disorder, Zhang et al. [12] demonstrated that healthy young adults with a family history of depression and increased CBF in the right amygdala were more susceptible to depression. A single-photon emission computed tomography (SPECT)-based study of brain perfusion in patients with schizophrenia revealed greater perfusion of the left amygdala in patients with hs-C-reactive protein levels ≥ 3 mg/L than in patients with hs-C-reactive protein levels < 3 mg/L [32]. The presence of greater cerebral perfusion in the amygdala might offer hemodynamic evidence supporting the involvement of the amygdala in CM pathophysiology.

Increased cerebral perfusion in the bilateral amygdala may reflect increased metabolic activity in brain tissue, which may be accompanied by structural changes and altered functional activity. A voxel-based morphometry study showed that gray matter volumes in the amygdala and putamen were greater in patients with CM than in patients with EM or HCs [10]. A systematic meta‑analysis of voxel-based morphometry studies regarding patients with migraine revealed similar results [33]. Additionally, a previous study of patients with migraine revealed that the amygdala volume was positively correlated with headache frequency in specific ranges [34]. These findings suggest that chronic pain alters structural plasticity in the brain because of repetitive headache episodes in migraine chronicization. However, a structural and functional MRI study by Chen et al. [11] did not demonstrate a significant difference in amygdala volume among HCs, patients with EM, and patients with CM. The disparate results of structural imaging studies may be related to the patients with various types of migraine.

Blood oxygenation level-dependent functional MRI (BOLD-fMRI) signals can indirectly reflect brain activity via neural activity-dependent changes in blood flow [35]. A previous study of BOLD-fMRI showed that functional connectivity between the amygdala and visceroceptive cortex is increased in patients with migraine, confirming the role of neurolimbic pain network dysfunction in CM chronicization [36]. Furthermore, Huang et al. [37] found that patients who had migraines without aura displayed enhanced effective connectivity from the left inferior frontal gyrus to the left amygdala. BOLD-fMRI and ASL techniques are used to assess hemodynamic information from different perspectives. BOLD-fMRI analysis may provide some support for the current results concerning hyperperfusion in the amygdala. Nevertheless, according to a literature review of resting-state functional connectivity MRI, a specific biomarker was not identified in migraine [38]. In future studies, functional connectivity and multi-delay pCASL imaging should be used to simultaneously construct a neurovascular coupling model of the amygdala and characterize the underlying pathogenesis of the amygdala in migraine.

There is increasing evidence that the amygdala is an important center for the emotional-affective dimension of pain and pain modulation in the brain [8]. Multiple nuclei in the amygdala are involved in its pain processing functions, including the lateral-basolateral complex (LA/BLA), central nucleus (CeA), and intercalated cell mass. The CeA receives inputs from spinal and trigeminal nociceptive neurons; it projects to various brain regions involved in pain perception, such as the hypothalamus, periaqueductal gray, and nucleus raphe [39–41]. Moreover, there is evidence that neurons in the amygdala regulate pain conduction by releasing glutamate and gamma-aminobutyric acid (GABA) neurotransmitters; these neurotransmitters may affect migraine onset [42–44]. Increased cerebral perfusion in the bilateral amygdala may be associated with neurotransmitter hyperactivation in the amygdala, which leads to increased neurotransmitter release from neurons in the amygdala and promotes migraine persistence [45].

The amygdala plays a key role in emotional expression; it is hyperactive in both anxious and depressive states [46]. A previous study revealed that epigenetic regulation of the calcitonin gene-related peptide (CGRP) gene was linked to anxiety- and depression-like behaviors [47]. Notably, CGRP receptor binding sites are present at high densities in the amygdala (CeA and BLA) [48]. Behavioral studies showed that stereotaxic administration of CGRP into the CeA of awake rats led to increased emotional responses and the onset of mechanical hypersensitivity [49]. Significant increases in CGRP levels have been identified in patients with CM, compared with HCs and patients with EM [45]. Therefore, the increased cerebral perfusion of bilateral amygdala in CM may be a primary factor involved in the development of comorbidities, such as anxiety and depression. A magnetoencephalography-based study showed that after negative emotional (negative/fearful) stimuli, the CM group had significantly greater amygdala activation, compared with the HC and EM groups, for M100 and M170 [50]. Another study revealed that greater depression severity led to an elevated risk of progression from EM to CM [51]. In the present study, we found that PHQ-9 and GAD-7 scores (representing depression and anxiety, respectively) [52] were higher in patients with CM than in patients with EM, which might indicate a higher tendency for patients with CM to develop psychiatric comorbidities.

In patients with migraine, chronic headache is particularly prevalent and can progress to MOH [53]. According to the ICHD-3, MOH constitutes a secondary headache caused by excessive use of acute headache medications for > 3 months [3]. Although ICHD-3 establishes a clear basis for the diagnosis of MOH, there remains debate in the medical community concerning whether MOH is a definitive distinct entity or a complication of primary headache disorders. In recent years, there has been increasing evidence that CM and MOH are closely related conditions [54–56]. Martelletti and other researchers have shown that MOH often results from the progression of a long-term chronic headache disorder, suggesting that MOH is a sequela or complication of CM [54]. Moreover, some researchers have argued that MOH and CM have similar underlying pathophysiologies that involve the sensitization of pain pathways in the brain [57]. Additionally, previous studies have demonstrated that therapies for CM are efficacious, regardless of MOH status [58, 59]. To validate the above findings, the present study explored whether medication overuse leads to altered cerebral perfusion of the bilateral amygdala in patients with CM. We observed no differences in cerebral perfusion of the bilateral amygdala in patients with CM, regardless of MOH status. This result suggests that MOH may arises from the progression of long-term CM and possesses shared hemodynamic characteristics with CM without MOH.

In this study, we used multi-delay pCASL MRI, a non-invasive and quantitative perfusion MRI technique, to identify changes in cerebral perfusion of the bilateral amygdala among patients with EM and patients with CM. Previous ASL studies generally used a single PLD time (e.g., 1.5–2 s) to estimate CBF [24, 30]. However, prolonged ATT may lead to CBF underestimation in brain tissue. Multi-delay pCASL imaging allows the acquisition of images with various PLD times. Compared with the existing single-delay ASL scans, multi-delay pCASL imaging has multiple potential advantages, including the inclusion of multiple hemodynamic parameters (ATT and CBF), improvements in CBF quantification accuracy, and better visualization of collateral flow via dynamic imaging [28]. In the present study, we encoded seven PLD times using seven effective label durations and acquired arrival-time-corrected CBF images. Studies of cerebrovascular disease have increasingly utilized multi-delay ASL methods to correct CBF values with respect to arrival time, highlighting the advantages of multi-delay ASL techniques in clinical applications [60, 61].

There were a few limitations in this study. First, it was a cross-sectional study that only included patients who had migraine without aura. Second, because of the limited sample size of patients with migraine in the attack phase, the cerebral perfusion characteristics between the attack and interictal phases were not distinguished in patients with migraine. However, the results suggested that cerebral perfusion parameters in the amygdala did not significantly differ between the attack and interictal phases in the Supplementary file of Tables S2-S3. In future studies, we plan to increase the sample size with a focus on changes in dynamic perfusion during various phases of the attack and interictal periods. Third, due to the limited spatial resolution of ASL imaging, it was impossible to accurately analyze the cerebral perfusion features in subregions of the amygdala. However, improving the spatial resolution will considerably decrease the signal-to-noise ratio and prolong the acquisition time. Further advances in spatial resolution and technical developments involving ASL techniques in vivo at high field strengths of 7 T MR are expected [62]. Moreover, due to the non-normality of some perfusion data, it is necessary to explore the clustering algorithm of perfusion or clinical variables in future research.

## Conclusion

The bilateral amygdala presents hyperperfusion in patients with CM, which might provide potential hemodynamic evidence in the neurolimbic pain network of CM.

### Supplementary Information

**Additional file 1: Table S1.** Demographics and clinical characteristics between CM without and with MOH. **Table S2.** The cerebral perfusion parameters of bilateral amygdala between interictal and attack periods in patients with CM. **Table S3.** The cerebral perfusion parameters of bilateral amygdala between interictal and attack periods in patients with EM. **Table S4.** The associations between the age and cerebral perfusion parameters of bilateral amygdala in three groups. **Table S5.** The associations between the BMI and cerebral perfusion parameters of bilateral amygdala in three groups. **Table S6.** Comparisons of age and BMI between males and females in three groups.

## Data Availability

Data can be made available upon request.

## Abbreviations

HC: Healthy control
EM: Episodic migraine
CM: Chronic migraine
MOH: Medication overuse headache
pCASL: Pseudo-continuous arterial labeling
PLD: Post-label delay
CBF: Cerebral blood flow
ATT: Arterial transit time
aCBV: Arterial cerebral blood volume
rs-fMRI: Resting-state functional magnetic resonance imaging
BOLD-fMRI: Blood oxygenation level-dependent functional MRI
HIT-6: Headache impact test-6
PHQ-9: Patient Health Questionnaire-9
GAD-7: Generalized anxiety disorder-7
PSQI: Pittsburgh sleep quality index
MNI: Montreal neurologic institute
AAL3: Automatic anatomical labeling atlas 3
CGRP: Calcitonin gene-related peptide
GABA: Glutamate and gamma-aminobutyric acid
