# Supplementary material for: Hyperperfusion of bilateral amygdala in patients with chronic migraine: an arterial spin-labeled magnetic resonance imaging study
Source: J Headache Pain. 2023 Oct 18;24(1):138. doi: 10.1186/s10194-023-01668-0 (PMC10583377; doi:10.1186/s10194-023-01668-0)
Supplement: Supplementary file 1 — Additional file 1: Table S1. Demographics and clinical characteristics between CM without and with MOH. Table S2. The cerebral perfusion parameters of bilateral amygdala between interictal and attack periods in patients with CM. Table S3. The cerebral perfusion parameters of bilateral amygdala between interictal and attack periods in patients with EM. Table S4. The associations between the age and cerebral perfusion parameters of bilateral amygdala in three groups. Table S5. The associations between the BMI and cerebral perfusion parameters of bilateral amygdala in three groups. Table S6. Comparisons of age and BMI between males and females in three groups. [file 10194_2023_1668_MOESM1_ESM.docx]

**Supplementary Results**

**Table S1** Demographics and clinical characteristics between CM without and with MOH

|  | **CM (n = 55)** | | ***P* value** |
| --- | --- | --- | --- |
|  | **CM without MOH (N=22)** | **CM with MOH (N=33)** |  |
| Age (years) | 49.00 (33.00-53.50) | 45.00 (34.50-51.00) | 0.414 |
| Female, n (%) | 17 (77.3) | 26 (78.8) | 0.894 |
| BMI (kg/m^2^) | 23.63 ± 2.92 | 23.02 ± 3.94 | 0.544 |
| Right-handers, n (%) | 22 (100.0) | 33 (100.0) | 1.000 |
| Unilateral headache, n (%) | 10 (45.5) | 15 (45.5) | 1.000 |
| Disease duration (years) | 19.14 ± 11.23 | 21.55 ± 10.46 | 0.420 |
| Headache frequency, days/month | 16.00 (15.00-30.00) | 30.00 (20.00-30.00) | **0.009**** |
| Headache intensity^a^ | 7.00 (5.50-8.00) | 8.00 (7.00-9.00) | **0.018*** |
| MIDAs (0-270) | 110.00 (58.00-179.00) | 100.00 (57.75-174.75) | 0.957 |
| HIT-6 score (36-78) | 66.00 (63.50-70.75) | 67.50 (64.00-72.75) | 0.571 |
| PHQ-9 score (0-27) | 10.00 (5.00-15.50) | 8.00 (3.00-17.00) | 0.679 |
| GAD-7 score (0-21) | 6.00 (2.50-13.50) | 6.00 (2.00-13.50) | 0.992 |
| PSQI score (0-21) | 10.30 ± 4.54 | 10.04 ± 4.85 | 0.851 |

*CM* chronic migraine, *MOH* medication overuse headache, *BMI* body mass index, *MIDAs* Migraine Disability Assessment Scale, *HIT-6* Headache Impact Test-6, *PHQ-9* Patient Health Questionnaire-9, *GAD-7* Generalized Anxiety Disorder-7, *PSQI* Pittsburgh Sleep Quality Index, *^a^* Headache intensity on a 0–10 numerical rating scale. * *P*＜0.05, ** *P*＜0.01.

**Table S2** The cerebral perfusion parameters of bilateral amygdala between interictal and attack periods in patients with CM

|  | **Sides** | **CM (n = 55)** | | ***P* value** |
| --- | --- | --- | --- | --- |
|  |  | **Interictal period (n=41)** | **Attack period (n=14)** |  |
| CBF (ml/100 g/min) | Left | 46.41 ± 9.62 | 46.75 ± 9.72 | 0.909 |
|  | Right | 42.71 (38.24-47.35) | 47.27 (40.14-56.04) | 0.105 |
| CBV (ml/100 g) | Left | 0.92 ± 0.23 | 0.90 ± 0.16 | 0.741 |
|  | Right | 0.86 (0.73-0.93) | 0.94 (0.81-1.11) | 0.147 |

*CM* chronic migraine, *CBF* cerebral blood flow, *aCBV* arterial cerebral blood volume.

**Table S3** The cerebral perfusion parameters of bilateral amygdala between interictal and attack periods in patients with EM

|  | **Sides** | **EM (n = 26)** | | ***P* value** |
| --- | --- | --- | --- | --- |
|  |  | **Interictal period (n=18)** | **Attack period (n=8)** |  |
| CBF (ml/100 g/min) | Left | 42.60 ± 9.62 | 46.13 ± 7.38 | 0.367 |
|  | Right | 41.33 ± 10.03 | 42.26 ± 9.45 | 0.827 |
| CBV (ml/100 g) | Left | 0.83 ± 0.20 | 0.93 ± 0.17 | 0.233 |
|  | Right | 0.83 ± 0.19 | 0.90 ± 0.16 | 0.361 |

*EM* episodic migraine, *CBF* cerebral blood flow, *aCBV* arterial cerebral blood volume.

**Table S4** The associations between the age and cerebral perfusion parameters of bilateral amygdala in three groups

|  | **Sides** | **HC (n = 26)** | | **EM (n = 26)** | | **CM (n = 55)** | |
| --- | --- | --- | --- | --- | --- | --- | --- |
|  |  | ***r*** | ***P*** | ***r*** | ***p*** | ***r*** | ***P*** |
| CBF (ml/100 g/min) | Left | -0.327 | 0.103 | -0.406 | 0.040* | -0.279 | 0.039* |
|  | Right | -0.194 | 0.341 | -0.377 | 0.057 | -0.287 | 0.033* |
| CBV (ml/100 g) | Left | -0.383 | 0.053 | -0.426 | 0.030* | -0.291 | 0.031* |
|  | Right | -0.233 | 0.252 | -0.292 | 0.148 | -0.197 | 0.150 |

*HC* healthy control, *EM* episodic migraine, *CM* chronic migraine. *CBF* cerebral blood flow, *aCBV* arterial cerebral blood volume. *Significant correlation between age and cerebral perfusion parameters at *P*<0.05.

**Table S5** The associations between the BMI and cerebral perfusion parameters of bilateral amygdala in three groups

|  | **Sides** | **HC (n = 26)** | | **EM (n = 26)** | | **CM (n = 55)** | |
| --- | --- | --- | --- | --- | --- | --- | --- |
|  |  | ***r*** | ***P*** | ***r*** | ***p*** | ***r*** | ***P*** |
| CBF (ml/100 g/min) | Left | -0.428 | 0.029* | -0.290 | 0.150 | -0.301 | 0.027* |
|  | Right | -0.430 | 0.028* | -0.251 | 0.216 | -0.311 | 0.022* |
| CBV (ml/100 g) | Left | -0.404 | 0.041* | -0.079 | 0.701 | -0.227 | 0.098 |
|  | Right | -0.183 | 0.371 | -0.133 | 0.517 | -0.218 | 0.114 |

*HC* healthy control, *EM* episodic migraine, *CM* chronic migraine. *BMI body mass index, CBF* cerebral blood flow, *aCBV* arterial cerebral blood volume. *Significant correlation between age and cerebral perfusion parameters at *P*<0.05.

**Table S6** Comparisons of age and BMI between males and females in three groups

|  | **Sex** | **Age (years)** | **BMI (kg/m^2^)** |
| --- | --- | --- | --- |
| HC (n = 26) | Male | 42.00 (39.00-53.00) | 22.86 (22.49-28.41) |
|  | Female | 37.00(28.00-46.00) | 21.83 (19.88-24.77) |
|  | *P* value | 0.306 | 0.120 |
| EM (n = 26) | Male | 37.57±11.28 | 24.21(22.23-25.83) |
|  | Female | 41.11±9.42 | 22.77 (20.70-27.12) |
|  | *P* value | 0.428 | 0.497 |
| CM (n = 55) | Male | 45.00 (33.00-56.00) | 25.65±4.16 |
|  | Female | 46.00 (34.00-52.00) | 22.66±3.13 |
|  | *P* value | 0.878 | 0.044* |

*HC* healthy control, *EM* episodic migraine, *CM* chronic migraine. *BMI body mass index*. *Significant correlation between male and female at *P*<0.05.
